# Application of Artificial Neural Networks for Prediction of Mechanical Properties of CNT/CNF Reinforced Concrete

**DOI:** 10.3390/ma14195637

**Published:** 2021-09-28

**Authors:** Sofija Kekez, Jan Kubica

**Affiliations:** Department of Structural Engineering, Faculty of Civil Engineering, Silesian University of Technology, Akademicka 5, 44-100 Gliwice, Poland; jan.kubica@polsl.pl

**Keywords:** concrete mix design methods, artificial neural networks, self-sensing concrete, CNT/CNF reinforced concrete

## Abstract

Prominence of concrete is characterized by its high mechanical properties and durability, combined with multifunctionality and aesthetic appeal. Development of alternative eco-friendly or multipurpose materials has conditioned improvements in concrete mix design to optimize concrete production speed and price, as well as carbon footprint. Artificial neural networks represent a new and efficient tool in achieving optimal concrete mixtures according to its intended function. This paper addresses concrete mix design and the application of artificial neural networks (ANNs) for self-sensing concrete. The authors review concrete mix design methods and the development of ANNs for prediction of properties for various types of concrete. Furthermore, the authors present developments and applications of ANNs for prediction of compressive strength and flexural strength of carbon nanotubes/carbon nanofibers (CNT/CNF) reinforced concrete using experimental results for the learning process. The goal is to bring the ANN approach closer to a variety of concrete researchers and possibly propose the implementation of ANNs in the civil engineering practice.

## 1. Introduction

Concrete is the most used construction material, primarily because of its high compressive strength and durability, but also for its impermeability and fire and corrosion resistance. Over the years, engineers have been developing different concretes for various and multiple purposes, with varieties spanning from ordinary concrete to specific types such as lightweight, high-performance (self-compacting, high-strength), green, or nano-reinforced concrete. Since processes of hydration and hardening are irreversible, any mistake in the design of the mixture may be costly in construction stage and hazardous in the exploitation stage. Therefore, optimal mix proportions and prediction of concrete properties have been studied over the past five decades, and many different methods have been developed to this end. Furthermore, in the past decade, machine learning (ML) methods emerged as a new tool of ensuring optimal concrete mixtures, and among many ML methods, artificial neural networks (ANNs) have been fairly successful in ensuring favorable results.

This paper reviews the development of ANNs for the purposes of concrete mix design and presents ANN models developed for prediction of compressive strength and flexural strength of CNT/CNF reinforced concrete. The goal of the authors is to bring the ANN approach closer to a variety of concrete behavior researchers and possibly propose the development and implementation of ANNs in mix design of nano-reinforced concrete.

### 1.1. Concrete Mix Design

Concrete mix design refers only to designed mixtures and does not imply prescribed, standard, or designated mixtures of concrete. It means that the designer specifies limiting values of needed key characteristics, assuming their effect on the properties of concrete. Design in the strict sense of the word is not possible; materials are variable in several aspects and many of their properties cannot be assessed quantitatively. Concrete properties measured in the fresh state are usually workability and slump, and in the hardened state compressive strength, permeability, and durability. Overall, the 28-day compressive strength is the critical design parameter for structural concrete. Concrete mix design implies one mixture for one set of properties, and every production that requires slightly different parameters comprehends a completely new design. Consequently, the entire process is relatively long due to trial mixing and testing of every specimen. It is a time-consuming procedure that not only increases the waste of material but also the cost of concrete production.

General classification includes analytical, semi-experimental, experimental, and statistical methods of concrete mix design. Analytical methods are used to reduce number of trial mixes to a minimum, rationalizing initial proportioning procedure into a systematic process based on detailed information about specific weight of components and formulae established from previously conducted testing [1]. In general, these methods are relatively quick and cost-friendly, but the main drawback is the uncertainty of results. Experimental methods are based on a trial-and-error process, where the biggest challenge is high number of effect variables affecting the response variables. The “one-factor-at-a-time” method’s main disadvantage is the lack of consideration of interaction between factors affecting the final parameters. Experimental methods give the most certainty in results; however, they are also the most time-consuming and expensive procedures. Statistical methods, also termed as factorial design methods, represent a step further where a set of trial mixes within a chosen range of proportions for each component is defined according to some statistical procedure. Afterward, trial mixes are conducted, test specimens are tested, and experimental results are analyzed using standard statistical methods. Although statistical methods require a certain amount of experimental work, their advantage is in the predictability with a higher level of certainty. However, the main issue remains the incapability of modeling complex nonlinear nature of the relationships between the mixture and the properties because underlying relationships are unknown.

Semi-experimental methods are based on combining the experimental models with various analytical tools such as machine learning (ML) methods. Development of more sophisticated non-parametric ML methods and growing availability of experimental datasets are opening opportunities to forecast compressive strength and other properties with higher accuracy and wider application range. This type of semi-experimental methods have shown to be useful in concrete mix design for prediction of various properties of fresh and hardened concrete.

### 1.2. Artificial Neural Networks

Machine learning belongs to the field of artificial intelligence, and it is used for prediction, i.e., classification that represents the prediction of the categorical value, or regression, which is the prediction of the numerical value [2]. Certain studies in concrete mix design that used ML were focused on modeling mixtures with particular ingredients, generating models that are predicting the compressive strength. Methods used in concrete mix design are artificial neural network (ANN), support vector machine (SVM), adaptive neuro-fuzzy inference system (ANFIS), random forest (RF), decision tree (DT), and more. Many investigations tend to make comparisons between ANNs and other ML methods to establish which is more efficient, as summarized in Table 1.

Artificial neural networks are computational structures that are trained to learn patterns from examples. The development of ANNs is inspired by the human brain, a biological neural network functioning based on communication between neurons. The theory of connectionism was first proposed in the 1940s to simulate processing of the human brain; however, at first, the idea was abandoned for many years until contribution of several researchers led a new interest in this subject. ANNs are used in a wide variety of problems such as recalling data, classifying patterns, performing general mapping from input to output patterns, grouping similar patterns, or solving constrained optimization problems [29]. Neural networks learn from parallel examples of input and output pairs and make generalizations [29,30,31], i.e., identifies causality between the input and the output through iterative training and using it to conduct forecast [4]. The ability to give correct or nearly correct responses to incomplete tasks and noisy or poor data makes ANNs a powerful tool for solving many civil engineering problems [5,32]. Additional advantages are unrestricted number of inputs and outputs [6,29], fast implementation [33], and user-friendliness [29]. Disadvantages are sensitivity to dataset, iterative process of determining the optimal structure, and hardware dependence [29]. ANNs are used in concrete mix design to predict optimal mix proportions or properties such as compressive and tensile strength [25,34,35,36,37], modulus of elasticity [38], slump [2,39,40,41], drying shrinkage [42], etc.

Feed-forward network topology represents dataflow from input to output units, where data processing can extend over multiple layers, but no feedback connections are present [43]. Mathematical functions that define behavior of every neuron are summation and activation function [44]. In general, obtaining a working ANN model is set in two stages: training and testing. The entire process of training can be simply described as follows. One input neuron represents one input variable. Each input is multiplied by corresponding weight after what the product is summed and applied to a transfer function to form output [45]. This scheme is mathematically, as follows:

(1) $$u_{k}=\sum (w_{km}*x_{m})$$

(2) $$y_{k}=f\left(u_{k}+b_{k}\right)$$

where *x*_1_, *x*_2_, …, *x*_*m*_ are the inputs, $w_{k1}$, $w_{k2}$, …, $w_{km}$ are the synaptic weights of the neuron *k*, *b_k_* is the bias for the neuron *k*, function *f* is an activation function, and $y_{k}$ is the output [30].

This process fits the architecture of a back-propagation algorithm. The “backpropagation learning rule” was established in 1985 as a solution to problems that were occurring with single layer or bilayer networks. It is considered a generalization of the delta rule [30,43,46] for multilayer networks, and the idea is to back-propagate the error of the outputs. After the training phase, testing of the network is necessary to determine efficiency and precision of the results. Validation is the intermittent procedure that is occasionally used to measure generalization and to halt training when generalization ceases to improve, indicating that testing has no further effect on training [7].

### 1.3. ANNs for Prediction of Concrete Material Behavior

The topic of ANNs in concrete mix design was primarily centered toward predictions of optimal mix proportions rather than predictions of the properties of concrete. Oh et al. [47] first discussed this topic, developing a predictive model for proportioning of concrete mixes However, as shown in [31], this approach, although somewhat useful, still implies development of a new model for every change of a constituent material. Investigations now focus on developing ANNs for prediction of the compressive strength of high-performance, green, ordinary, or other types of concrete. Table 2 gives an overview on the mixtures, showing blends used for compressive strength tests. Several works focused on the technical problems such as determination of the optimal algorithm for compressive strength predictions, and others focused on factors that may influence the quality of results of both experimental tests and predictive models.

Lee [48] showed an extensive study on the efficacy of ANNs in prediction of concrete strength, where five independent models were developed with a staggering 73 input variables and as many as seven outputs. Chopra et al. [23,24,49] developed ANN models for compressive strength prediction, focusing on execution of the model itself. The authors in [49] used seven different algorithms to determine the optimal one for their dataset. Furthermore, they observed efficacy of the ANN model compared to genetic programming [24], decision tree, and random forest models [23]. Golafshani et al. [27] compared the results of the ANN and ANFIS models and continued further by optimizing the models with Grey Wolf Optimizer to establish prediction models for plain and high-performance concrete.

**Table 2:** Various types of concrete mixtures investigated using ANNs.

Aggregate Type/ Binder Type	Plain Cement	Silica Fume	Blast Furnace Slag	Fly Ash	Micro and Nano-Silica	Metakaolin
Standardized Fine/Coarse Aggregate	[8,15,16,19,23,28,29,33,47,49,50,51,52]	[11,12,21,32,41,53,54]	[6,7,8,9,12,14,15,17,20,27,55,56,57,58,59]	[6,7,8,9,12,14,15,17,20,24,31,41,53,54,55,56,57]	[25,56,60]	[32,44]
Recycled Concrete Aggregate	[3,5,26,30,37,61]			[62,63]		
Recycled Rubber Aggregate	[22,64]	[18]				
Basalt Powder	[64]		[38]			
Limestone Crushed/ Sand	[2,65,66]	[10]				
Rice Husk Ash	[37]	[10,67]		[67]	[67]	[67]
Artificial Aggregate	[38,68]	[10]		[69]	[69]	[69]

Conversely, research work such as that of Dantas et al. [30] focused on the impact of various constituent materials, in this case, construction and demolition waste. An investigation by Yaman et al. [31] focused on highly flowable self-compacting concrete and developed ANN models with two datasets, where the first model comprised all six outputs and the second was in the form of a multi-input-single-output model. Regarding an environmentally friendly approach, Elevado et al. [62] presented completely green concrete, replacing Portland cement with fly ash and coarse aggregate with waste ceramic tiles. ANNs were used for compressive strength predictions, and the results showed this is a possible alternative for traditional concrete.

Several investigations were conducted from building sites to make ANN models more pragmatic and useful in realistic conditions. In situ works imply a lack of controllability of environmental conditions, which influence concrete in both a fresh and hardened state. Therefore, testing of field concrete is a more challenging task compared to laboratory, but the dataset is far more extensive and insightful. Namyong et al. [52] presented statistical investigation of field concrete based on 1442 results from 59 different mixtures. The authors [52] used relatively large dataset to establish regression equations for predictions of compressive strength. DeRousseau et al. [50] evaluated the efficacy of ANNs and other ML methods for prediction of compressive strength of field-placed concrete using two datasets from both field and laboratory. This work confirmed that the accurate prediction of compressive strength of field concrete is achieved with ML models trained on field concrete data, and that by using hybrid training data predictive performance of laboratory concrete models might be significantly improved. Furthermore, the work of Young et al. [15] included probably the most extensive dataset ever used in this type of investigation. ANN models were based on more than 10,000 data tuples obtained from building sites and the laboratory testing.

Although most research work is focused on predicting the compressive strength, there are notable works handling other properties of concrete. Predictions of mechanical properties of hardened concrete such as flexural strength [34] for modified zeolite additive mortar, or [36] for hybrid composites, elastic modulus of recycled aggregate concrete [70], Poisson’s ratio of lightweight concrete [71], fatigue strength [72], freeze-thaw durability [73], and electrical property prediction [74], showed to be useful. There have also been investigations focused on the properties of fresh concrete such as drying shrinkage [42], structural properties such as chloride permeability [75,76] and diffusivity [77], air void content [78], as well as the dependency of compressive strength on the concrete microstructure [79].

Finally, reviewing the application of ANNs for prediction of concrete properties may provide conclusions. A number of hidden layers should be kept low. More hidden layers prolong the learning process and often cause false positive result. Algorithms, usually Levenberg–Marquardt, resilient BP, BFGS quasi-Newton, and Polak–Ribiere conjugate gradient, are chosen according to the nature of the data and type of the output. Size of the dataset influences the algorithm performance and thus the total error. It has been repeatedly shown that the Levenberg–Marquardt algorithm corresponds best with a medium-sized dataset containing a few hundred data tuples. Activation functions are by default non-linear, mostly sigmoid (logistic) and hyperbolic tangent since these functions coincide with material behavior of concrete. Testing tuples must be diverse to give the best evaluation of the model, but at the same time kept within a realistic confidence interval.

## 2. Prediction of Properties of Self-Sensing Concrete Using ANNs

This work attempts to establish a working ANN model for prediction of compressive strength and flexural strength of ordinary concrete reinforced by carbon nanotubes (CNTs) or carbon nanofibers (CNFs). All models are developed, trained, and tested using Matlab Neural Fitting tool. Following, we describe the procedure of establishing optimal parameters of the ANN models for each concrete property separately and combined. The workflow follows typical schematic of extracting and collecting of the data from literature, preprocessing, and finally application of the dataset as an input for the ANN [80], and it is described in detail in the following.

### 2.1. Training Parameters of ANN Models

Parameters describing the basis of a neural network are architecture, algorithm, and activation function. Architecture, or topology, of the ANN model refers to the number of layers and neurons within each layer. The function of a hidden layer is to detect and establish relationship between inputs and outputs. In this work, all ANN models use “shallow” architecture, meaning that there is only one hidden layer. Size of the hidden layer is problem specific and depends on the training patterns. Namely, there is no established rule for selecting the number of neurons for each hidden layer. It must be sufficiently low to ensure generalization of the network, but if it is too low, the network will not be able to learn the relationships from the data and generalize to new data [4,7,81]. Many studies have related the number of hidden neurons to the number of input and output variables (Table 3) and training patterns; however, these rules cannot be generally accepted [7,45,81] and dogmatically followed. Although trying several architectures and selecting the optimal one is a relatively long process, it is performed to determine the stability and efficacy of the network [45].

This research work uses a varied number of neurons, calculated according to Equation (3). Except for the dependency between the number of input and hidden neurons given by the literature, additional dependency is investigated to establish if there is an effect to the performance of the model.

(3) $$N_{h}=N_{i};\;2N_{i}+1;\;3\cdot N_{i}$$

Back-propagation feed-forward multiple layer neural networks are trained with many different training algorithms, depending on the specific problem as well as the size of the network and the training dataset. The most used algorithm within a concrete mix design is the Levenberg–Marquardt algorithm due to its speed and robustness [4], and it is also used in this work for all ANN models. It is the fastest training algorithm for moderately sized networks with up to few hundred weights [30,44].

Activation function represents a “mathematical gate”, which data “goes through” on its way to the next layer of the neural network. In other words, output signal of the neuron relates to input via the activation function [32]. Choice of the activation function may strongly influence the complexity and performance of the ANN [44,82,83]. Non-linear activation functions are used in concrete mix design. The most used is a unipolar sigmoid; however, certain researchers use bipolar sigmoid or hyperbolic tangent [29,44], although common practice includes several activation functions between individual layers within one network.

Learning process for all ANN models in this work is provided by the Levenberg–Marquardt training algorithm with unipolar sigmoid activation function and linear transfer function. Other parameters of the model performance are maximum number of epochs at 1000; training momentum is 10^9^; learning rate is 10^−6^; and 6 cross-validation checks during learning.

### 2.2. Datasets

A prerequisite for successful functioning of an ANN is the use of extensive and reliable dataset capable of appropriate training [44]. Form, content, and size of the dataset has great effect to the computation of appropriate outputs. Format of the dataset is important because the way data is presented to the network affects the training process. Both input and output variables must be normalized, usually within the range of [0,1] as it is done in this work, or scaled to the range of (−1,+1) or (0.1,0.9). When preprocessing of data is finished, the set is divided to subsets for training, validation, and testing. Training subset contains the highest percentage of the total amount of data, usually from 65–80, or 90 percent. The rest is then left to be used for testing or is divided between testing and validation subsets. Data tuples are shuffled randomly between subsets to avoid any possible effect on the training algorithm [4]. In this work, datasets are divided into subsets with ratios 70/20/10; 80/10/10; 80/15/5; and 85/10/5 for training/testing/validation.

More data i.e., bigger batch size, does not necessarily lead to a better network. Although a richer dataset leads to better generalization, if the quality of the data is not at a high level, batch size itself does not make a difference for network performance.

In this work, datasets represent the collection of experimental data given in the literature [84,85,86,87,88,89,90,91,92,93,94,95,96,97,98,99,100,101,102,103,104,105,106,107,108,109,110,111]. Experimental investigations were chosen according to the content of the mixture, type of the nanofiller, and fabrication and testing procedures. All samples fabricated in the given experimental procedures were tested to confirm the proper dispersion of the nanofiller. Three datasets with different number of data tuples are used, each used to train and test twelve ANN models. Datasets are given by the output parameter, namely, COMP gives compressive strength, FLEX flexural strength, and C+F gives both compressive and flexural strength of CNT/CNF reinforced concrete mixtures. All mixtures contain either CNT or CNF additions, and that there are no hybrid nano-reinforcements. Table 4 gives the outline of each dataset, and Table 5 summarizes input and target output parameters of each dataset.

### 2.3. ANN Models

ANN models are developed and divided by their respective dataset, since the number of hidden neurons depends on the number of input neurons, which varies by the dataset. For each dataset, 12 ANN models are developed and divided into four subgroups according to the subset ratio, and each subgroup includes three ANN models with different number of hidden neurons, according to Equation (3). There are 36 ANN models in total, which are summarized in Table 6. The nomenclature of the models is given in the form X_NNtr_val_tes-Nh, where X is the name of the dataset, tr is training subset percentage, val is validation subset percentage, tes is testing subset percentage, and Nh is the number of hidden neurons. For example, COMP_NN70_10_20-20 represents a neural network (NN) model with dataset COMP, with subset ratio 70/10/20 for training/validation/testing, and with 20 hidden neurons in the one hidden layer.

### 2.4. Results

The ultimate goal of any training procedure is to minimize the mean square error (MSE) and mean absolute error (MAE) and maximize the coefficient of regression R. The iterations run until no improvement in MSE and MAE is found. Accuracy of the results is usually presented by the value of R, meaning that in case of a perfect fit between output and target value, this value would be equal to 1. In general, every phase of the network development demands a trial-and-error procedure to check suitability and stability of the network. After the initial training of the ANN models, response values R and MSE indicate the efficiency of the model. Response values for all ANN models are given in Table 7, where the best results are bolded and used in further analysis. Table 7 gives the results of total values of regression coefficient R, as generated by Matlab tool. There is also the number of epochs, showing how fast a generalization has been achieved. This number may imply the possibility of false positive results if it is too low to be considered that the network made enough iterations and was able to learn and establish the final values of weights and bias.

**Figure 1:**
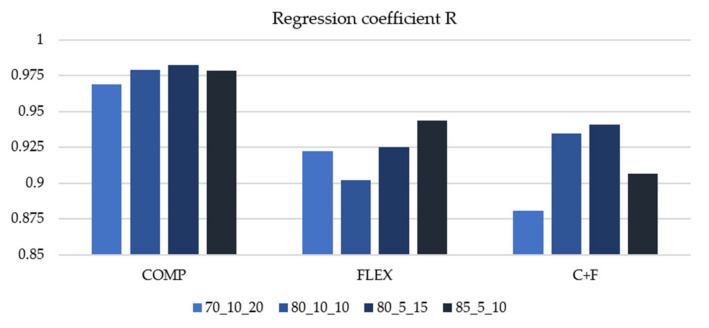
Histogram of regression coefficients for ANN models.

**Figure 2:**
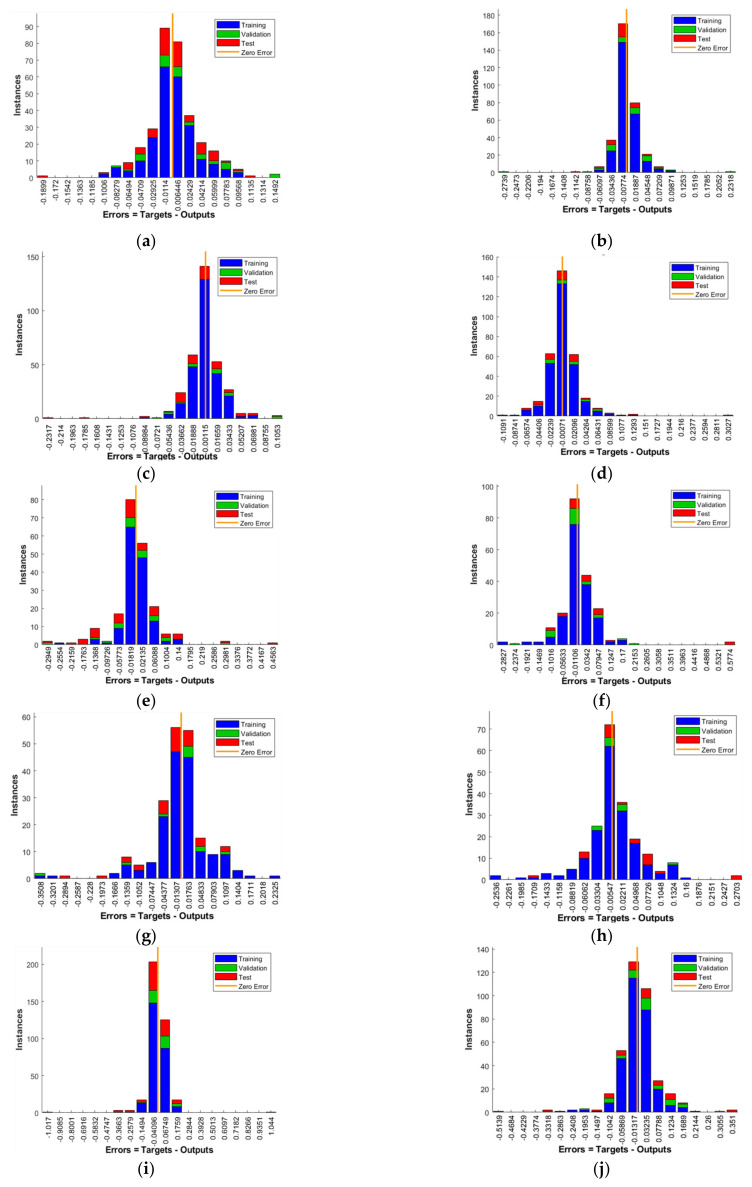

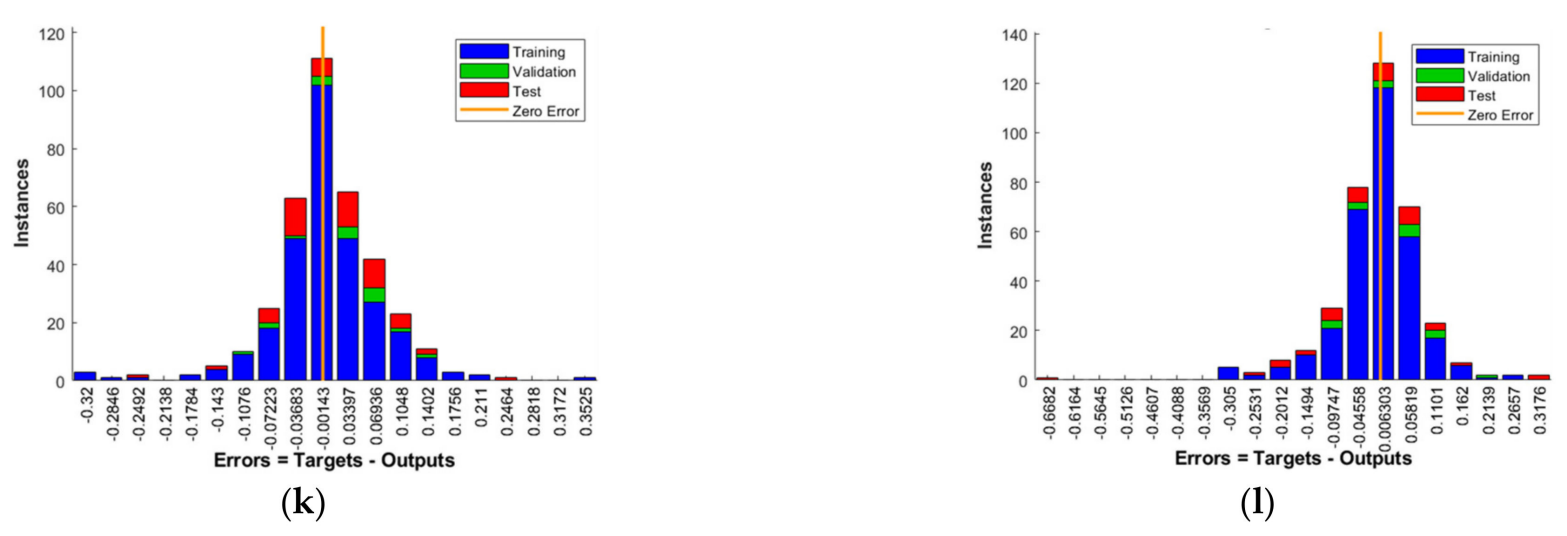
Error histogram for model: (**a**) COMP_NN70_10_20-41; (**b**) COMP_NN80_10_10-60; (**c**) COMP_NN80_5_15-20; (**d**) COMP_NN85_5_10-41; (**e**) FLEX_NN70_10_20-48; (**f**) FLEX_NN80_10_10-48; (**g**) FLEX_NN80_5_15-16; (**h**) FLEX_NN85_5_10-16; (**i**) C+F_NN70_10_20-33; (**j**) C+F_NN80_10_10-11; (**k**) C+F_NN80_5_15-11; (**l**) C+F_NN85_5_10-33.

As it may be seen in Table 7, ANN models gave satisfactory results with regression coefficient R values higher than 0.80 and higher than 0.85 for the best models within each subgroup, as it is shown in Figure 1. Since the differences in regression coefficients within each group are minute, we will observe other pointers of models’ efficiency. Except for regression coefficient R, useful indicator of network behavior may be the error histogram which shows zero-centered Gauss curve at optimal learning trend. Error histograms of the best models from each subgroup are shown in Figure 2.

Figure 2 gives the distribution of error (x-axis) over instances (y-axis), and it shows that the most regular distribution of error is obtained for models (a) COMP_NN70_10_20-41, (g) FLEX_NN80_5_15-16, and (k) C+F_NN80_5_15-11. In other words, subset ratios with relatively more training data, and equal or relatively close numbers of hidden and input neurons, present the architecture with most favorable results. Since there was an investigation of Nh = 3Ni number of hidden neurons, these models showed comparable behavior and results, and it may imply favorable behavior for a smaller number of input neurons. It can only be an assumption that it would give better results if the number of tuples was higher, or if another activation function was used. Results of training, testing, validation, and total regression coefficients for the optimal ANN models is given in Table 8.

## 3. Discussion and Conclusions

Concrete mix design requires extensive knowledge of many expert issues. Inherently, obtaining concrete with appropriate parameters ensures reliable use during the prescribed exploitation period. In construction of massive structures, or commercial and residential buildings, concrete mix design assures that required parameters are achieved while keeping the costs at the necessary minimum. The ANN approach gives the possibility to freely adjust and change mix proportions according to exposure to certain materials and needed type of concrete. The construction speed and quality control may be significantly increased while decreasing the costs and carbon footprint by using ANNs to determine and predict properties of fresh and hardened concrete. Additionally, ANNs represent an appealing tool for modeling complex systems because of features such as efficiency, generalization, and simplicity. 

This paper assesses the predictions of compressive strength and flexural strength of CNT/CNF reinforced concrete. To this purpose, 36 models were developed using three different datasets. One dataset uses both target values, compressive and flexural strength, and the remainder have a singular property as the target value. The models were trained using Matlab Neural Fitting application. After training, validation, and testing, it may be concluded that all models show satisfactory behavior with the given topologies. In addition, all three variants of hidden neurons used here correspond to this type of neural network in achieving successful training of the network. Moreover, initial research shows that mechanical properties of CNT/CNF reinforced concrete can be successfully predicted using the described ANN models. Out of 36 models in total, models COMP_70_10_20-41, FLEX_80_5_15-16, and C+F_80_5_15-11 exhibited optimal results, most uniform error distribution, and therefore, overall most favorable behavior. The regression coefficients for training, testing, and validation stage are high and the scattering of the results is relatively small for these models. Thus, the results confirmed the correctness of the adopted models and calculations. It may be concluded that tested network topology, algorithm, and activation function give satisfactory results in assessing the problems of the mechanical properties of CNT/CNF reinforced concrete composite material. This research work shows that further research in this direction may give promising results and can move further toward developing a novel method of concrete mix design.

## Figures and Tables

**Table 1:** Investigations which compare ANNs to other methods for prediction of concrete behavior.

Prediction Method	Reference
MLR–Multiple linear regression	[3,4,5,6,7,8,9,10,11,12,13]
SVM–Support vector machine	[2,6,13,14,15,16,17,18]
ANFIS–Adaptive neuro-fuzzy inference system	[5,10,11,18,19,20]
FL–Fuzzy logic	[2,21,22]
RF–Random forest	[2,17,23]
DT–Decision tree	[2,15,23]
GP–genetic programming	[18,24,25]
M5PMT–M5P Model tree	[9,26,27]
Salp swarm algorithm	[27,28]
CART–Classification and regression tree	[12]

**Table 3:** Empirical recommendations for determining number of neurons in the first hidden layer.

$2\cdot N_{i}$
$N_{i}+N_{o}$
$0.75\cdot N_{i}$
$2N_{i}+1$
$N_{i}$
$\left(N_{i}+N_{o}\right)/2$

*N_i_*, number of input nodes; *N_o_*, number of output nodes.

**Table 4:** Outline of each dataset.

Dataset	Nomenclature	Data Tuples	Input Neurons	Output Neurons
1	COMP	329	20	1
2	FLEX	207	16	1
3	C+F	185	11	2

**Table 5:** Input neurons for each dataset and their minimum/maximum values.

	COMP			FLEX			C+F	
#	Neuron	Min/Max	#	Neuron	Min/Max	#	Neuron	Min/Max
1	CEM	317.61/1875 kg/m^3^	1	CEM	317.61/1578.95 kg/m^3^	1	CEM	317.61/1578.95 kg/m^3^
2	WAT	121.6/789.48 kg/m^3^	2	WAT	142/789.48 kg/m^3^	2	WAT	142/789.48 kg/m^3^
3	FA	0/1994.4 kg/m^3^	3	FA	0/1994.4 kg/m^3^	3	FA	0/1994.4 kg/m^3^
4	CA	0/1284 kg/m^3^	4	CA	0/1284 kg/m^3^	4	CA	0/1284 kg/m^3^
5	SPL	0/27.27 kg/m^3^	5	SPL	0/27.27 kg/m^3^	5	SPL	0/27.27 kg/m^3^
6	CNT	0/2 wt%	6	CNT	0/0.5 wt%	6	CNT	0/0.5 wt%
7	CNF	0/2.5 wt%	7	CNF	0/2 wt%	7	CNF	0/2 wt%
8	CEM-CLASS	42.5/52.5	8	CEM-CLASS	42.5/52.5	8	CEM-CLASS	42.5/52.5
9	FUNCT	0/1 (no/yes)	9	FUNCT	0/1 (no/yes)	9	FUNCT	0/1 (no/yes)
10	C_S-A	0/1 (no/yes)	10	C_S-A	0/1 (no/yes)	10	DEM-AGE	24/48 h
11	C_S-B	0/1 (no/yes)	11	C_S-B	0/1 (no/yes)	11	AGE	3/120 days
12	C_S-C	0/1 (no/yes)	12	C_S-C	0/1 (no/yes)	1	OUTPUT 1	19.8/97.2 MPa
13	C_S-D	0/1 (no/yes)	13	C_S-D	0/1 (no/yes)	2	OUTPUT 2	2.18/16.4 MPa
14	C_S-E	0/1 (no/yes)	14	C_S-E	0/1 (no/yes)			
15	C_S-F	0/1 (no/yes)	15	DEM-AGE	18/48 h			
16	C_S-G	0/1 (no/yes)	16	AGE	3/120 days			
17	C_S-H	0/1 (no/yes)	1	OUTPUT	2.18/16.4 MPa			
18	C_S-I	0/1 (no/yes)						
19	DEM-AGE	24/48 h						
20	AGE	3/120 days						
1	OUTPUT	4.4/152 MPa						

**Table 6:** Summary of models, their architecture, and subset division.

#	Model	Dataset	Input Neurons	Hidden Neurons	Output Neurons	Training % (#)	Validation % (#)	Testing % (#)
1	COMP_NN70_10_20-20	COMP	20	20	1	70% (230)	10% (33)	20% (66)
2	COMP_NN70_10_20-41	COMP	20	41	1	70% (230)	10% (33)	20% (66)
3	COMP_NN70_10_20-60	COMP	20	60	1	70% (230)	10% (33)	20% (66)
4	COMP_NN80_10_10-20	COMP	20	20	1	80% (263)	10% (33)	10% (33)
5	COMP_NN80_10_10-41	COMP	20	41	1	80% (263)	10% (33)	10% (33)
6	COMP_NN80_10_10-60	COMP	20	60	1	80% (263)	10% (33)	10% (33)
7	COMP_NN80_5_15-20	COMP	20	20	1	80% (264)	5% (16)	15% (49)
8	COMP_NN80_5_15-41	COMP	20	41	1	80% (264)	5% (16)	15% (49)
9	COMP_NN80_5_15-60	COMP	20	60	1	80% (264)	5% (16)	15% (49)
10	COMP_NN85_5_10-20	COMP	20	20	1	85% (280)	5% (16)	10% (33)
11	COMP_NN85_5_10-41	COMP	20	41	1	85% (280)	5% (16)	10% (33)
12	COMP_NN85_5_10-60	COMP	20	60	1	85% (280)	5% (16)	10% (33)
13	FLEX_NN70_10_20-16	FLEX	16	16	1	70% (145)	10% (21)	20% (41)
14	FLEX_NN70_10_20-33	FLEX	16	22	1	70% (145)	10% (21)	20% (41)
15	FLEX_NN70_10_20-48	FLEX	16	48	1	70% (145)	10% (21)	20% (41)
16	FLEX_NN80_10_10-16	FLEX	16	16	1	80% (165)	10% (21)	10% (21)
17	FLEX_NN80_10_10-33	FLEX	16	22	1	80% (165)	10% (21)	10% (21)
18	FLEX_NN80_10_10-48	FLEX	16	48	1	80% (165)	10% (21)	10% (21)
19	FLEX_NN80_5_15-16	FLEX	16	16	1	80% (166)	5% (10)	15% (31)
20	FLEX_NN80_5_15-33	FLEX	16	22	1	80% (166)	5% (10)	15% (31)
21	FLEX_NN80_5_15-48	FLEX	16	48	1	80% (166)	5% (10)	15% (31)
22	FLEX_NN85_5_10-16	FLEX	16	16	1	85% (176)	5% (10)	10% (21)
23	FLEX_NN85_5_10-33	FLEX	16	22	1	85% (176)	5% (10)	10% (21)
24	FLEX_NN85_5_10-48	FLEX	16	48	1	85% (176)	5% (10)	10% (21)
25	C+F_NN70_10_20-11	C+F	11	11	2	70% (129)	10% (19)	20% (37)
26	C+F_NN70_10_20-23	C+F	11	23	2	70% (129)	10% (19)	20% (37)
27	C+F_NN70_10_20-33	C+F	11	33	2	70% (129)	10% (19)	20% (37)
28	C+F_NN80_10_10-11	C+F	11	11	2	80% (147)	10% (19)	10% (19)
29	C+F_NN80_10_10-23	C+F	11	23	2	80% (147)	10% (19)	10% (19)
30	C+F_NN80_10_10-33	C+F	11	33	2	80% (147)	10% (19)	10% (19)
31	C+F_NN80_5_15-11	C+F	11	11	2	80% (147)	5% (9)	15% (28)
32	C+F_NN80_5_15-23	C+F	11	23	2	80% (147)	5% (9)	15% (28)
33	C+F_NN80_5_15-33	C+F	11	33	2	80% (147)	5% (9)	15% (28)
34	C+F_NN85_5_10-11	C+F	11	11	2	85% (157)	5% (9)	10% (19)
35	C+F_NN85_5_10-23	C+F	11	23	2	85% (157)	5% (9)	10% (19)
36	C+F_NN85_5_10-33	C+F	11	33	2	85% (157)	5% (9)	10% (19)

**Table 7:** Response values of ANN models.

#	Model Nomenclature	Regression Coefficient R	Mean Squared Error MSE	Epoch
1	COMP_NN70_10_20-20	0.95267	0.00195	11
2	**COMP_NN70_10_20-41**	**0.9688**	**0.00107**	**10**
3	COMP_NN70_10_20-60	0.96502	0.00151	11
4	COMP_NN80_10_10-20	0.96367	0.000979	12
5	COMP_NN80_10_10-41	0.98091	0.000766	23
6	**COMP_NN80_10_10-60**	**0.9791**	**0.000415**	**29**
7	**COMP_NN80_5_15-20**	**0.98256**	**0.000391**	**32**
8	COMP_NN80_5_15-41	0.93784	0.00367	8
9	COMP_NN80_5_15-60	0.96813	0.001488	9
10	COMP_NN85_5_10-20	0.97202	0.001193	27
11	**COMP_NN85_5_10-41**	**0.97858**	**0.000681**	**24**
12	COMP_NN85_5_10-60	0.97274	0.001157	11
13	FLEX_NN70_10_20-16	0.91543	0.005345	15
14	FLEX_NN70_10_20-33	0.89097	0.007012	9
15	**FLEX_NN70_10_20-48**	**0.92254**	**0.001995**	**24**
16	FLEX_NN80_10_10-16	0.87775	0.00877	9
17	FLEX_NN80_10_10-33	0.89732	0.00707	9
18	**FLEX_NN80_10_10-48**	**0.90198**	**0.00396**	**23**
19	**FLEX_NN80_5_15-16**	**0.92508**	**0.005**	**20**
20	FLEX_NN80_5_15-33	0.86375	0.008407	8
21	FLEX_NN80_5_15-48	0.87383	0.005773	10
22	**FLEX_NN85_5_10-16**	**0.94388**	**0.00378**	**32**
23	FLEX_NN85_5_10-33	0.93602	0.00461	18
24	FLEX_NN85_5_10-48	0.91121	0.00533	10
25	C+F_NN70_10_20-11	0.86913	0.009495	10
26	C+F_NN70_10_20-23	0.90129	0.0076	12
27	**C+F_NN70_10_20-33**	**0.88072**	**0.004027**	**18**
28	**C+F_NN80_10_10-11**	**0.93468**	**0.003361**	**27**
29	C+F_NN80_10_10-23	0.95102	0.003566	21
30	C+F_NN80_10_10-33	0.83489	0.005389	16
31	**C+F_NN80_5_15-11**	**0.94066**	**0.005587**	**19**
32	C+F_NN80_5_15-23	0.93937	0.00511	16
33	C+F_NN80_5_15-33	0.95118	0.004076	12
34	C+F_NN85_5_10-11	0.83851	0.01363	10
35	C+F_NN85_5_10-23	0.88078	0.008397	10
36	**C+F_NN85_5_10-33**	**0.90683**	**0.00636**	**10**

**Table 8:** Regression coefficients of the models with best performance.

#	Model Nomenclature	Training	Testing	Validation	Total
1	COMP_NN70_10_20-41	0.9764	0.95837	0.95823	0.9688
2	FLEX_NN80_5_15-16	0.93545	0.90633	0.87768	0.92508
3	C+F_NN80_5_15-11	0.94032	0.93642	0.97165	0.94066

## Data Availability

The data presented in this study are openly available in reference number [84,85,86,87,88,89,90,91,92,93,94,95,96,97,98,99,100,101,102,103,104,105,106,107,108,109,110,111].
